# Optimal blood pressure for patients with end‐stage renal disease following coronary interventions

**DOI:** 10.1111/jch.14325

**Published:** 2021-07-15

**Authors:** Ya‐Ling Yang, Su‐Chan Chen, Cheng‐Hsueh Wu, Shao‐Sung Huang, Wan Leong Chan, Shing‐Jong Lin, Chia‐Yu Chou, Jaw‐Wen Chen, Pan Ju‐Pin, Min‐Ji Charng, Ying‐Hwa Chen, Tao‐Cheng Wu, Tse‐Min Lu, Pai‐Feng Hsu, Po‐Hsun Huang, Hao‐Min Cheng, Chin‐Chou Huang, Shih‐Hsien Sung, Yenn‐Jiang Lin, Hsin‐Bang Leu

**Affiliations:** ^1^ Division of Cardiology Department of Medicine Cardinal Tien Hospital Taiwan ROC; ^2^ Department of Medicine School of Medicine National Yang‐Ming Chiao University Taipei Taiwan ROC; ^3^ Division of Cardiology Department of Medicine Taipei Veterans General Hospital Taipei Taiwan ROC; ^4^ Healthcare and Management Center Taipei Veterans General Hospital Taipei Taiwan ROC; ^5^ Institute of Clinical Medicine and Cardiovascular Research Center National Yang‐Ming University Taipei Taiwan ROC

**Keywords:** blood pressure, coronary artery disease, end‐stage renal disease, percutaneous coronary intervention

## Abstract

Hypertension is a frequent manifestation of chronic kidney disease but the ideal blood pressure (BP) target in patients with coronary artery disease (CAD) with end‐stage renal disease (ESRD) (eGFR < 15 ml/min/1.73m^2^) still unclear. The authors aimed to investigate the ideal achieved BP in ESRD patients with CAD after coronary intervention. Five hundred and seventy‐five ESRD patients who had undergone percutaneous coronary interventions (PCIs) were enrolled and their clinical outcomes were analyzed according to the category of systolic BP (SBP) and diastolic BP (DBP) achieved. The clinical outcomes included major cardiovascular events (MACE) and MACE plus hospitalization for congestive heart failure (total cardiovascular (CV) event).The mean systolic BP was 135.0 ± 24.7 mm Hg and the mean diastolic BP was 70.7 ± 13.1 mm Hg. Systolic BP 140–149 mm Hg and diastolic BP 80–89 mm Hg had the lowest MACE (11.0%; 13.2%) and total CV event (23.3%; 21.1%). Patients with systolic BP < 120 mm Hg had a higher risk of MACE (HR: 2.01; 95% CI: 1.17–3.46, *p* = .008) than those with systolic BP 140–149 mm Hg. Patients with systolic BP ≥ 160 mm Hg (HR: 1.84; 95% CI, 3.27–1.04, *p* = .04) and diastolic blood BP ≥ 90 mm Hg (HR: 2.19; 95% CI: 1.15–4.16, *p* = .02) had a higher risk of total CV event rate when compared to those with systolic BP 140–149 mm Hg and diastolic BP 80–89 mm Hg. A J‐shaped association between systolic (140–149 mm Hg) and diastolic (80–89 mm Hg) BP and decreased cardiovascular events for CAD was found in patients with ESRD after undergoing PCI in non‐Western population.

## 1 INTRODUCTION

High blood pressure (BP) is an important risk factor for cardiovascular disease, and BP is significantly related to the risk of mortality and morbidity.^1^ In the general population, BP values are correlated with cardiovascular (CV) risk starting from 115/75 mm Hg, and each 2 mm Hg reduction of DBP carries a 5% MI and 10% CV death risk reduction, indicating the importance of BP reduction for risk reduction.^2^ Interestingly, the benefit of maintaining a lower BP target is not always observed in all patients. Adequate diastolic BP is crucial for coronary perfusion, and a lower BP target is not consistently beneficial for all coronary artery disease (CAD) patients, suggesting that BP targets should be considered individually for CVD. Our previous study reported that systolic BP < 120 mm Hg and ≥ 160 mm Hg or diastolic BP < 70 mm Hg was associated with a higher risk of CV events in CAD patients after coronary intervention,^3^ providing the optimal BP target for CAD patients after percutaneous coronary intervention (PCI) in the Asian population. Among these CAD patients, those with poor renal function were especially important and attracted our attention. Renal failure or end‐stage renal disease (ESRD) is a special disease entity among chronic kidney disease (CKD)s, and recommended treatment targets for the general population usually fail in this high‐risk group. For example, statins are suggested to reduce cardiovascular disease (CVD) mortality for CAD patients, including patients with CKD. However, the beneficial effect of statins was not observed in patients with ESRD or patients who underwent dialysis.^4^ The complex interactions between factors such as malnutrition, inflammation, oxidative stress, vascular calcification, and rapidly progressing atherosclerosis may be responsible for the lack of effect of statins in patients with advanced renal failure, suggesting that the ESRD group is a special group of interest and that more clinical evidence is needed to provide better care for this high‐risk group.

Additionally, maintaining adequate renal perfusion is believed to play an important role in caring for patients with simultaneously combined CKD and CAD. Although most clinical trials did not enroll patients with poor renal function, current guidelines still suggest that patients with CKD should be considered a high‐risk group and that aggressive BP control is recommended to reduce future risk.^5^, ^6^, ^7^ However, the recommended BP value for patients with ESRD has not been established to date, and few studies have reported the optimal BP target for the ESRD population that simultaneously has CAD. Our current study aimed to investigate the optimal BP target for CAD patients with ESRD. The study aimed to investigate the effect of achieved BP on clinical outcomes in a cohort of ESRD patients who underwent successful coronary intervention.

## 2 METHODS

This larger retrospective, single‐center observational study included participants with symptomatic CAD who received a PCI between July 2006 and December 2015 at the Taipei Veteran General Hospital, Taiwan. In brief, CAD diagnosis included positive results on the stress test, a history of angina with ECG ischemic changes, myocardial infarction, or angina symptoms with a significant stenosis lesion on coronary computed tomography angiography (CCTA). All patients who received PCI either with coronary stenting or balloon angioplasty were screened.

Patients with CKD stage V, defined as an eGFR < 15 ml/min/1.73m^2^,^8^ or patients who received dialysis were eligible for enrollment. The estimated glomerular filtration rate (eGFR) was calculated using a modified Modification of Diet in Renal Disease (MDRD) equation based on the Chinese population.^9^ All procedures were conducted in accordance with the Declaration of Helsinki and were approved by the Ethics Committee and Independent Review Board of the Taipei Veterans General Hospital and written informed consent was obtained from all study participants. For the dead patient, the informed consent came from kin and/or legally authorized representative (LAR). All patients should give their written informed consent before enrollment.

After enrollment, PCI procedures and treatment strategies were recorded. Baseline characteristics, including sex, age, history of hypertension, diabetes, hyperlipidemia, smoking, family history of premature CAD, and cerebral vascular disease, were collected from every patient. Furthermore, biochemical data describing renal function, lipid profiles, and medications were also collected by trained study nurses and qualified cardiologists. Blood pressure was measured by a well‐trained nurse with an electronic BP monitor according to the ACC/AHA Guideline suggestion for recording BP in adults^10^ and the Taiwan Hypertension Society (THS) recommendations.^11^ BP was recorded in the outpatient clinic, and the mean achieved systolic BP (SBP) and diastolic BP (DBP) were calculated by serial BP recording during the follow‐up period.

During follow‐up, the primary outcome, major adverse cardiovascular events (MACE), which included cardiovascular death, nonfatal myocardial infarction, and nonfatal stroke, was recorded. The secondary outcome was the total cardiovascular event (total CV event), which was a composite of the MACE plus hospitalization for congestive heart failure. Myocardial infarction was confirmed in patients presenting with ischemic symptoms with elevated serum cardiac enzyme levels and/or characteristic ECG changes. Ischemic stroke was confirmed as an obstruction within a brain blood vessel with imaging evidence by either MRI or CT scan and a new neurological deficit lasting for at least 24 h. The protocol for CV event follow‐up was performed as previously described.^12^ Reporting of the study conforms to STROBE statement along with references to STROBE statement and the broader EQUATOR guidelines (Simera and coworkers January 2010 issue of EJCI).

Categorical and continuous variables were expressed as the mean ± standard deviation or frequency (percentage). Between various BP groups, the chi‐square test or Fisher's exact test was used for comparing the continuous data and the categorical variables were analyzed by one‐way analysis of variance (ANOVA). The clinical outcomes were presented as overall percentages and expressed as proportions with a 95% confidence interval (CI). The prognostic difference and event‐free survival rate between groups of patients with various maintained BP levels were analyzed using the Kaplan–Meier method based on the log‐rank test. Hazard ratios (HRs) from the Cox regression model were used to analyze each outcome. *p*‐values < .05 were considered significant. In addition to crude hazard ratios (HRs), adjusted HRs were estimated after adjustment for potential confounding factors, including age, sex, history of hypertension, diabetes, and smoking habit. All analyses were performed using the statistical package SPSS for Windows (Version 22.0, SPSS Inc., Chicago, IL, USA)

## 3 RESULTS

A total of 575 patients (age 70.63 ± 12.58 years, 343 males [57.9%]) with ESRD who underwent successful coronary intervention were enrolled. The baseline characteristics of the participants according to the achieved SBP and DBP categories are presented in **Tables** **1** and **2**, respectively. Patients maintaining higher SBP tended to be younger, female and have more comorbidities, including hypertension and diabetes mellitus. Regarding DBP, patients with higher DBP tended to be younger, more hypertensive, and used more statins, beta‐blockers (BB), calcium channel blockers (CCB), and vasodilator agents.

**TABLE 1:** Baseline characteristics of chronic renal disease and achieved systolic blood pressure (*n* = 575)

	All (*n* = 575)	SBP < 120 (*n* = 165)	SBP = 120‐129 (*n* = 97)	SBP = 130‐139 (*n* = 83)	SBP = 140‐149 (*n* = 73)	SBP = 150‐159 (*n* = 64)	SBP ≧ 160 (*n* = 93)	*p*
Age (year)	70.63±12.58	73.14±12.04	70.22±13.81	70.01±12.21	71.35±11.58	70.78±12.81	66.52±12.27	.004
Male (*n* (%))	343 (59.7)	112 (67.9)	64 (66)	56 (67.5)	40 (54.8)	30 (46.9)	41 (44.1)	<.001
Diabetes (*n* (%))	340 (59.1)	82 (49.7)	46 (47.4)	53 (63.9)	44 (60.3)	46 (71.9)	69 (74.2)	<.001
Hypertension (*n* (%))	503 (87.5)	130 (78.8)	79 (81.4)	77 (92.8)	65 (89)	64 (100)	88 (94.6)	<.001
Smoking (*n* (%))	189 (32.9)	57 (34.5)	32 (33)	28 (33.7)	22 (30.1)	20 (31.3)	30 (32.3)	.989
CHF (*n* (%))	177 (30.8)	55 (33.3)	26 (26.8)	27 (32.5)	19 (26)	26 (40.6)	24 (25.8)	.296
BMI (kg/m^2^)	24.64 ± 3.92	24.63 ± 4.03	24.57±3.70	25.15±4.00	24.02 ± 3.54	25.03 ± 3.08	24.55 ± 4.60	.579
Systolic BP (mm Hg)								
During PCI	137.82±17.63	124.13±15.39	133.78±13.64	139.55±15.81	146.64±12.91	146.49±15.31	159.45±23.00	<.001
Mean SBP maintained	132.03±16.55	109.99±8.44	125.03±3.12	134.83±2.85	144.36±3.07	154.63±2.74	166.80±6.96	<.001
Creatinine (mg/dl)	5.12±3.44	4.28±2.94	4.94±3.46	4.94±3.63	5.45±3.65	5.57±3.08	6.35±3.73	<.001
Total cholesterol (mg/dl)	164.26±45.51	155.00±41.34	163.60±52.30	164.00±44.88	165.57±36.15	172.51±47.45	172.91±47.89	.043
HDL‐C (mg/dl)	40.08±12.83	137.22±84.51	120.44±61.40	151.32±94.51	140.8±70.10	147.73±94.72	157.47±98.94	.066
LDL‐C (mg/dl)	96.02±34.94	37.57±12.30	40.54±11.52	40.84 ± 13.21	40.09 ± 11.55	43.30 ± 12.73	40.76 ± 15.08	.086
Triglyceride (mg/dl)	141.46±85.33	90.66±34.40	97.68±37.79	92.57 ± 31.57	97.90 ± 30.36	100.60±34.55	101.40±38.22	.175
Glucose (mg/dl)	131.88±51.81	130.85±49.65	129.13±43.67	131.96±50.23	129.09±64.42	132.30±51.44	137.32±55.54	.958
CAD severity								
SVD (*n* (%))	83 (14.4)	23 (13.9)	15 (15.5)	13 (15.7)	11 (15.1)	6 (9.4)	15 (16.1)	.958
DVD (*n* (%))	159 (27.7)	48 (29.1)	24 (24.7)	24 (28.9)	19 (26)	17 (26.6)	27 (29)	
TVD (*n* (%))	328 (57)	93 (56.4)	58 (59.8)	46 (55.4)	42 (57.5)	40 (62.5)	49 (52.7)	
DES number (*n*)	2.03 ± 1.18	1.98 ± 1.29	1.86 ± 1.03	2.12 ± 1.09	2.13 ± 1.20	2.03 ± 1.26	2.12 ± 1.18	.821
DES diameter (mm)	2.94 ± 0.35	2.95 ± 0.39	2.98 ± 0.35	2.89 ± 0.32	2.90 ± 0.31	2.92 ± 0.32	2.98 ± 0.33	.707
DES length (mm)	25.53 ± 6.28	25.00 ± 6.40	24.44 ± 6.61	27.37 ± 5.99	25.75 ± 6.50	26.44 ± 6.97	24.92 ± 4.96	.168
BMS number (*n)*	1.67 ± 0.83	1.90 ± 0.99	1.45 ± 0.69	1.66 ± 0.72	1.78 ± 0.85	1.57 ± 0.73	1.50 ± 0.73	.083
BMS diameter (mm)	3.13 ± 0.52	3.12 ± 0.45	3.22 ± 0.55	3.23 ± 0.69	2.99 ± 0.37	3.14 ± 0.66	3.07 ± 0.44	.459
BMS length (mm)	22.51 ± 6.64	23.03 ± 6.56	23.58 ± 6.63	21.37 ± 5.95	22.06 ± 6.97	21.59 ± 6.68	22.31 ± 7.36	.735
Statin (*n* (%))	264 (45.9)	56 (33.9)	39 (40.2)	39 (47)	37 (50.7)	40 (62.5)	53 (57)	<.001
ACEi/ARB (*n* (%))	197 (34.3)	38 (23)	27 (27.8)	30 (36.1)	33 (45.2)	27 (42.2)	42 (45.2)	.001
Ca^+^ channel blocker (*n* (%))	232 (40.3)	33 (20)	27 (27.8)	35 (42.2)	37 (50.7)	45 (70.3)	55 (59.1)	<.001
Beta‐blocker (*n* (%))	280 (48.7)	55 (33.3)	49 (50.5)	43 (51.8)	44 (60.3)	36 (56.3)	53 (57)	<.001
Anti‐angina (*n* (%))	298 (51.8)	68 (41.2)	43 (44.3)	45 (54.2)	43 (58.9)	42 (65.6)	57 (61.3)	.002

Values are *n* (%) or mean ± SD.

Anti‐angina agents includes nitrate/nicorandil.

*Abbreviations*: BMI, body weight index; HDL, high‐density lipoprotein; LDL, low density lipoprotein; DES, drug eluting stent; BMS, bare metal stent; ACEi, angiotensin converting enzyme inhibitor; ARB, angiotensin II receptor blocker.

**TABLE 2:** Baseline characteristics of chronic renal disease and achieved diastolic blood pressure (*n* = 575)

	ALL (*n* = 575)	DBP <70 mm Hg (*n* = 280)	DBP = 70–79 mm Hg (*n* = 164)	DBP = 80–89 mm Hg (*n* = 76)	DBP≧90 mm Hg (*n* = 54)	*p*
Age (year)	70.66 ± 12.54	75.21 ± 10.11	69.76 ± 12.27	63.12 ± 12.84	60.39 ± 12.86	<.001
Male (*n* (%))	342 (59.6)	164 (58.6)	106 (64.6)	42 (55.3)	30 (55.6)	.422
Diabetes (*n* (%))	340 (59.2)	161 (57.5)	102 (62.2)	42 (55.3)	35 (64.8)	.544
Hypertension (*n* (%))	502 (87.5)	230 (82.1)	150 (91.5)	73 (96.1)	49 (90.7)	.002
Smoking (*n* (%))	188 (32.8)	88 (31.4)	52 (31.7)	27 (35.5)	21 (38.9)	.684
CHF (*n* (%))	177 (30.8)	87 (31.1)	51 (31.1)	20 (26.3)	19 (35.2)	.748
BMI (kg/m^2^)	24.64 ± 3.93	24.50 ± 3.96	24.94 ± 3.82	24.84 ± 3.91	24.18 ± 4.15	.545
Diastolic BP (mm Hg)						
During PCI	71.75±10.39	67.71±8.55	76.61±8.34	86.41±7.81	90.26±7.85	<.001
Mean DBP maintained	67.53±9.70	61.43±5.97	74.00±2.79	83.83±2.83	95.17±3.96	<.001
Creatinine (mg/dl)	5.12 ± 3.46	4.76 ± 2.99	5.09 ± 3.62	5.58 ± 3.99	6.48 ± 4.04	.005
Total cholesterol (mg/dl)	164.31 ± 45.46	159.12 ± 42.56	164.43 ± 44.62	177.21 ± 51.10	170.82 ± 49.95	.018
HDL‐C (mg/dl)	40.01 ± 12.72	39.23 ± 12.63	40.57 ± 11.67	41.76 ± 12.61	39.43 ± 16.37	.457
LDL‐C (mg/dl)	96.14 ± 34.89	92.44 ± 34.07	95.97 ± 30.66	105.32 ± 39.02	102.35 ± 42.94	.028
Triglyceride (mg/dl)	141.71 ± 85.31	138.86 ± 88.94	141.53 ± 79.32	147.70 ± 82.05	148.02 ± 91.73	.826
Glucose (mg/dl)	131.88 ± 51.81	130.50 ± 49.17	134.06 ± 51.81	132.67 ± 57.03	130.20 ± 58.76	.946
CAD severity						
SVD	36 (12.9)	24 (14.6)	14 (18.4)	8 (14.8)	36 (12.9)	.925
DVD	81 (29)	45 (27.4)	21 (27.6)	11 (20.4)	81 (29)	
TVD	160 (57.3)	94 (57.3)	40 (52.6)	34 (63)	160 (57.3)	
DES number (n)	2.03 ± 1.18	2.04 ± 1.23	2.08 ± 1.11	1.83 ± 1.06	2.04 ± 1.34	.706
DES diameter (mm)	2.94 ± 0.35	2.93 ± 0.37	2.93 ± 0.31	2.97 ± 0.30	3.02 ± 0.36	.616
DES length (mm)	25.53 ± 6.28	26.10 ± 6.41	24.68 ± 6.49	25.06 ± 5.50	26.16 ± 5.67	.323
BMS number (*n*)	1.69 ± 0.86	1.75 ± 0.94	1.73 ± 0.80	1.56 ± 0.77	1.45 ± 0.67	.403
BMS diameter (mm)	3.13 ± 0.52	3.21 ± 0.57	3.02 ± 0.43	3.06 ± 0.46	3.11 ± 0.56	.170
BMS length (mm)	22.44 ± 6.62	22.17 ± 6.53	21.85 ± 6.15	23.85 ± 7.65	23.66 ± 7.10	.477
Statin (*n* (%))	264 (46)	108 (38.6)	85 (51.8)	48 (63.2)	23 (42.6)	.001
ACEi/ARB (*n* (%))	65 (11.3)	27 (9.6)	21 (12.8)	11 (14.5)	6 (11.1)	.594
Ca^+^ channel blocker (*n* (%))	232 (40.4)	89 (31.8)	74 (45.1)	44 (57.9)	25 (46.3)	<.001
Beta‐blocker (*n* (%))	280 (48.8)	112 (40)	96 (58.5)	42 (55.3)	30 (55.6)	.001
Anti‐angina (*n* (%))	297 (51.7)	129 (46.1)	100 (61)	41 (53.9)	27 (50)	.024

Values are n (%) or mean ± SD.

Anti‐angina agents includes nitrate/nicorandil.

*Abbreviations*: BMI, body weight index; HDL, high‐density lipoprotein; LDL, low density lipoprotein; DES, drug eluting stent; BMS, bare metal stent; ACEi, angiotensin converting enzyme inhibitor; ARB, angiotensin II receptor blocker.

**FIGURE 1:**
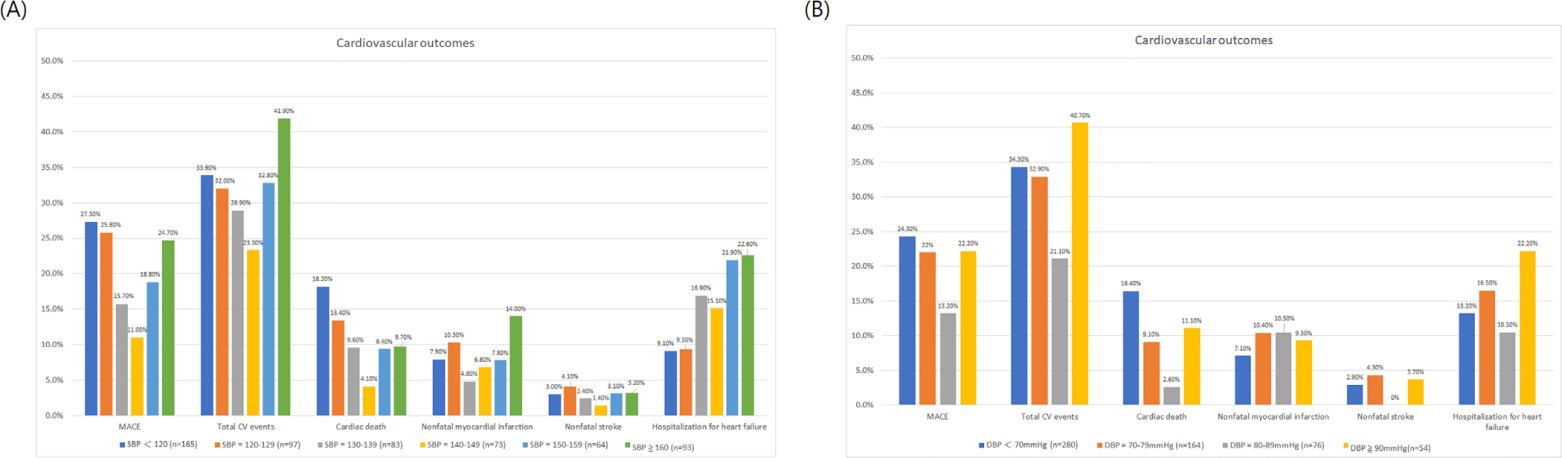
Cardiovascular outcomes in kidney failure patients with different blood pressure range. (A) Systolic blood pressure subgroups; (B) Diastolic blood pressure subgroups

Clinical follow‐up was carried out with all patients for a mean period of 65.6 months. During this time, there were 66 cardiovascular deaths, 175 cardiac nonfatal myocardial infarctions, 64 nonfatal strokes, and 287 hospitalizations for heart failure. **Figure** **1** shows the incidence of adverse events such as nonfatal MI, nonfatal stroke, cardiac death, MACE, hospitalization for CHF, and total CV events among various BP categories. In the SBP categories, the 140–149 mm Hg systolic BP group had a lower incidence of cardiac death (4.1%), MACE (11.0%), total CV event (23.3%) and nonfatal stroke (1.4%) compared with patients in the achieved SBP < 120 mm Hg, 120–129 mm Hg, 130–139 mm Hg, 150–159 mm Hg and ≥160 mm Hg groups (**Figure** **1** **A**). Regarding the CHF hospitalization incidence, there was a linear association between SBP values and the incidence of hospitalization for heart failure.

**FIGURE 2:**
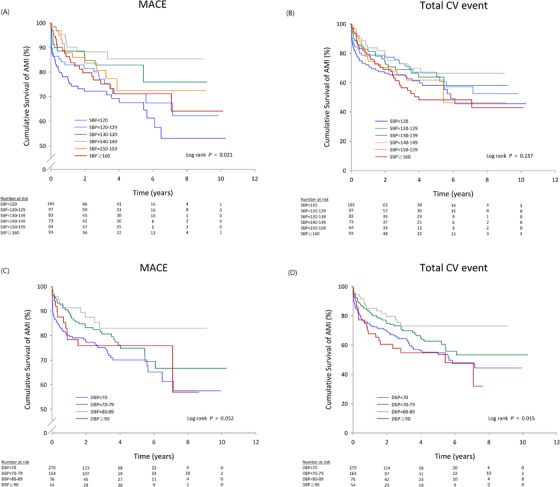
Kaplan‐Meier survival curve analysis about renal failure in systolic blood pressure subgroup showing (A) Major adverse cardiac events; (B) Total cardiovascular event, and in diastolic blood pressure subgroup; (C) Major adverse cardiac events; (D) Total cardiovascular event

In the diastolic BP categories, patients who maintained a diastolic BP of approximately 80–89 mm Hg had a lower incidence of MACE (13.3%), total CV event (21.1%), cardiac death (2.6%), nonfatal stroke (0%), and HF hospitalization (10.5%) (**Figure** **1** **B**) than patients in the other diastolic BP groups. Kaplan–Meier curves were used to assess the occurrence of future adverse events according to the categories of SBP and DBP maintained in the ESRD patients who underwent coronary PCI (**Figure** **2**). There was a significant difference in MACE (**Figure** **2** **A**) amid various SBP and DBP categories and in the total CV event (**Figure** **2** **D**) among the DBP groups (*p* < .05).

**FIGURE 3:**
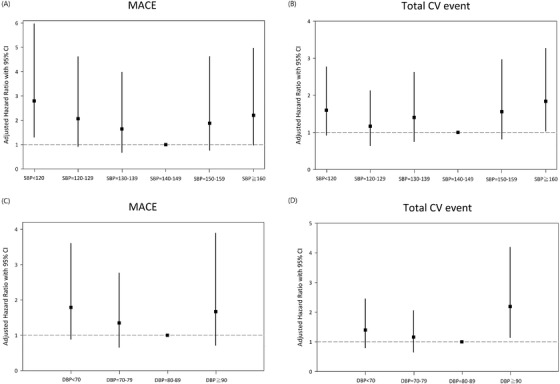
The association between BP and risk of poor outcome in ESRD patients following PCI. (A) MACE and systolic BP; (B) Total CV event and systolic BP; (C) MACE and diastolic BP; (D) Total CV and diastolic BP. *Adjusted with age and sex, history of hypertension, diabetes, and smoking

**Table** **3** shows the risk of primary (MACE; *includes cardiac death, nonfatal MI, nonfatal stoke) and secondary events (total CV event: *MACE pluse hospitalization for CHF) according to the BP categories after adjusting for **age, sex, hypertension, diabetes, and smoking habit. There was a J‐curve association between the achieved SBP and future adverse events. Compared with those in the 140–149 mm Hg SBP (reference group), patients with high SBP (> 160 mm Hg) were associated with a higher risk of MACE (HR: 2.21, 95% CI: 0.98–4.96) and total CV event (HR: 1.84, 95% CI: 1.04–3.27). The similar J‐curve result was found in cardiac death, nonfatal myocardial infraction, and nonfatal stroke (**Table S1**). Patients with lower SBP (< 120 mm Hg) were also associated with a higher risk of MACE (HR: 2.80, 95% CI: 1.31–5.96) and total CV event (HR: 1.60, 95% CI: 0.92–2.77) (**Figure** **3** **)**. The same phenomenon appears in different age groups (age < 70 years and ≥ 70 years). In both groups, diastolic BP found similar U‐shape result was found in MACE and total CV event. For those > 70 years, U‐shape was still found in total CV event, but not so significant for the MACE (Table S2).

**TABLE 3:** Clinical outcome in chronic renal disease patients according to systolic and diastolic blood pressure subgroups

		Systolic BP			Diastolic BP	
		Crude HR	Adjusted HR		Crude HR	Adjusted HR
MACE	<120 mm Hg	2.99 (1.41–6.35)	2.80 (1.31–5.97)	< 70 mm Hg	2.20 (1.16–4.39)	1.78 (0.88–3.58)
	120–129 mm Hg	2.25 (1.02–5.00)	2.06 (0.92–4.61)	70–79 mm Hg	1.58 (0.78–3.18)	1.35 (0.66–2.76)
	130–139 mm Hg	1.41 (0.59–3.41)	1.64 (0.68–3.98)	80‐89 mm Hg	Referent	Referent
	140–149 mm Hg	Referent	Referent	≧ 90 mm Hg	1.80 (0.78–4.12)	1.67 (0.72–3.88)
	150–159 mm Hg	1.68 (0.69–4.12)	1.88 (0.77–4.62)			
	≧ 160 mm Hg	2.01 (0.94–4.69)	2.21 (0.98–4.96)			
Total CV event	<120 mm Hg	1.76 (1.02–3.02)	1.60 (0.92–2.68)	< 70 mm Hg	2.03 (1.19–3.44)	1.39 (0.80–2.43)
	120–129 mm Hg	1.27 (0.70–2.30)	1.17 (0.64–2.12)	70‐79 mm Hg	1.49(0.85–2.60)	1.16(0.65–2.05)
	130–139 mm Hg	1.23 0.66–2.30)	1.4 (0.75–2.62)	80‐89 mm Hg	Referent	Referent
	140–149 mm Hg	Referent	Referent	≧ 90 mm Hg	2.29 (1.20–4.35)	2.19(1.15–4.18)
	150–159 mm Hg	1.43 (0.76–2.72)	1.56 (0.82–2.96)			
	≧ 160 mm Hg	1.73 (0.98–3.01)	1.84 (1.04–3.27)			

Major adverse cardiac Event (MACE) includes cardiac death, nonfatal MI, nonfatal stoke; Total major events includes MACE plus hospitalization for CHF.

^a^ Nonfatal MI: nonfatal myocardial infraction; stroke: nonfatal stroke; Heart failure: Hospitalization for heart failure.

^b^ Adjusted with age and sex, history of hypertension, diabetes, and smoking.

## 4 DISCUSSION

In the present investigation, we provide novel insights into the interplay of BP targets and future adverse events in ESRD patients who underwent coronary intervention. Our study's main finding was that SBP seems to have a J‐curve association with adverse events in which SBP of approximately 140–149 mm Hg had the lowest incidence rates of MACE, CV total event, cardiac death, and nonfatal stroke compared to the higher or lower SBP groups. DBP had similar findings; a diastolic BP of approximately 80–89 mm Hg had the lowest incidence rates of MACE, CV total event, cardiac death, nonfatal stroke and hospitalization for heart failure. After adjusting for comorbidities, an SBP of approximately 140–149 mm Hg and a DBP of approximately 80–89 mm Hg were associated with a lower risk of developing future MACE and total CV events.

Renal failure or ESRD is a special subgroup of CKD. Blood pressure before and after dialysis was especially susceptible to changes in fluid volume and electrolyte change in the body. Volume overload and sodium excess were the major mechanisms of high BP in dialysis patients.^10^ Other pathogenic mechanisms, including sympathetic nervous system activation, the renin‐angiotensin‐aldosterone system,^13^ arterial stiffness,^14^ and endothelial dysfunction,^15^ also contributed to BP modulation. ESRD patients usually have hypertension^16^ and this hypertension is very difficult to control due to BP fluctuations caused by intravascular volume changes and, usually, long‐term advanced atherosclerosis in the system vasculature. Despite the few randomized trials, the 2018 ESC/ESH hypertension guidelines suggested maintaining the SBP treatment threshold below 130–139 mm Hg in CKD patients.^5^ Although the optimal BP target is undetermined in patients with ESRD, poorly controlled BP is considered linked to worse clinical outcomes and associated with high all‐cause and cause‐specific mortality among dialysis patients.^17^, ^18^ Several studies showed the J‐ or U‐curve phenomenon (with excessively low systemic pressures) between BP values and future outcome^19^ and risk of vascular access thrombosis in dialysis patients.^20^ Dana C. Miskulin and coworkers^21^ compared standardized predialysis systolic BP with intensive control (110–140 mm Hg) or standard control (155–165 mm Hg) in a small randomized control trial. In that study, the intensive control group had a higher risk of recurrent hospitalization, vascular access thrombosis, intradialytic hypotension (SBP < 90 mm Hg), muscle cramps, and nausea/vomiting complications. Thierry Hannedouche and coworkers^17^ reported a U‐shaped association of systolic BP (lowest HR was 165 mm Hg) and an L‐shaped association of diastolic BP with all‐cause mortality. Similarly, there was a U‐shaped association of systolic BP (lowest HR was 157 mm Hg) and diastolic BP (lowest HR was 90 mm Hg) with cardiovascular mortality after reviewing 9333 chronic hemodialysis patients in France.^17^ All of the above studies suggested that the optimal BP target with the lowest future risk was different from the target in patient with CKD or the general population.

In our current study, we first clearly demonstrated the optimal BP maintained in ESRD patients combined with advanced CAD. All patients have received successful coronary intervention for their CAD. To our interest, there have also been many debates about optimal BP maintenance in patients with CAD. In an international cohort study conducted by Vidal‐Petiot and coworkers, it appears that systolic BP less than 120 mm Hg and diastolic BP less than 70 mm Hg increased cardiovascular risk, including cardiovascular death, myocardial infarction, or stroke, in patients with stable CAD.^22^ Our previous study showed a similar result: non‐Western, stable CAD patients who achieved systolic BP < 120 mm Hg and ≥160 mm Hg or diastolic BP < 70 mm Hg had increased cardiovascular events.^3^ The J‐curve hypothesis suspects decreased coronary perfusion with overtreatment of diastolic BP, which may cause myocardial ischemia and increase coronary events risk, especially in patients with CAD. The SBP associated with the lowest adverse events in dialysis CAD patients was 140–149 mm Hg, which is different from the range of 120–139 mm Hg observed in our previous CAD population.

The reason why the optimal BP for dialysis CAD patients is higher than that for other CAD patients may have several explanations. First, ESRD/dialysis patients are more vulnerable to volume changes. Low BP may deteriorate blood flow to vital organs, leading to hypoperfusion of the cerebral circulation, reduced myocardial perfusion, and worse residual kidney function.^23^ Mac Ewen C and coworkers reported a significant correlation between a decline of 10 mm Hg in mean arterial pressure from baseline and an increase of 3% in ischemic events.^24^ In addition, BP control is usually very difficult in dialysis patients, and BP fluctuation is frequently observed. Hypertension treatment may affect patients’ eGFR fluctuation by 10–20%, and adequate BP must be maintained to avoid complications such as intradialytic hypotension.^25^ Low BP also causes low cardiac output status, which increases mortality in dialysis patients. Burton JO and coworkers reported that decreases in systolic BP may cause myocardial stunning and regional wall motion abnormalities. Ultrafiltration volume was an independent determinant associated with myocardial stunning, suggesting that maintaining relatively high BP may be better in dialysis patients.^26^

Interestingly, there was a linear (ie, not a J‐curve) association between BP and the incidence of hospitalization for heart failure. This was reasonable because all dialysis patients needed dialysis to maintain output balance. Higher BP may represent unstable sodium‐fluid homeostasis. Volume overload, excess sodium, and a decompensated state that could deteriorate heart function, further leading to acute decompensated heart failure.^27^ Therefore, intensive BP control exerted a strong protective effect in preventing hospitalization for heart failure outcome.^28^ This may explain why there was no J‐curve phenomenon observed in the relationship between BP values and the incidence of hospitalization for heart failure in our study.

This study has limitations that should be mentioned. First, we have ever grouped those CAD patients according to six SBP and four DBP categories. Some BP groups have few cases (*n* < 3) and it was difficult to make a conclusion based on limited cases. However, we still can found that patients whose SBP between 140 and 149 plus DBP between 80 and 89 had the lowest incidence of future MACE and total CV events. Therefore, a larger‐scale population is needed to verify the current study findings. Second, we used in‐hospital and outpatient clinic visit BP recordings, not home BP measurements. The bias between hospital‐visit BP measurements and home BP measurements cannot be excluded. Third, our study was from a single‐center observational registry. Patients were enrolled and followed up regularly for clinical events in the outpatient clinics of the medical centers or teaching hospitals, which could not reflect the referral nature of the practice at this tertiary referral medical center in Taiwan. Finally, cancer survivors had a higher risk of cardiovascular diseases such as deep vein thrombosis, heart failure, arrhythmia, pericarditis, and usually had poor outcome. In our study, there were 15 CAD patients with cancer history enrolled in our study, and no cardiac death was found among these patients. Because few cancer patients enrolled in our current study and limited events were identified, the cancer effect cannot be evaluated. We provide evidence of a J‐curve association between SBP and DBP and adverse cardiovascular events in CAD patients who underwent dialysis after PCI. The SBP and DBP associated with the lowest future MACE were 140–149 mm Hg and 80–90 mm Hg, respectively. For the relationship between heart failure incidence and BP values, no J‐curve association was observed. A larger‐scale population‐based study is needed to confirm our observations.

## CONFLICT OF INTEREST

The Author(s) declare(s) that there is no conflict of interest.

## AUTHOR CONTRIBUTIONS

A consortium has been organized to conduct this study. H.‐B. Leu, and Y.‐L. Yang, contributed to the study design. Y.‐L. Yang contributed to infrastructure design and technician training for this project. All investigators of this study (C.‐H. Wu, S.‐S. Huang, W.L. Chan, Shing‐Jong Lin, Chia‐Yu Chou, Jaw‐Wen Chen, Pan Ju‐Pin, Min‐Ji Charng, Ying‐Hwa Chen, Tao‐Cheng Wu, Tse‐Min Lu, Pai‐Feng Hsu, Po‐Hsun Huang, Hao‐Min Cheng, Chin‐Chou Huang, Shih‐Hsien Sung, Yenn‐Jiang Lin) are in charge of collecting clinical informatics and tissue sample in hospital. H.‐B. Leu, S.‐C. Chen, Y.‐L. Yang contributed to data analysis and data interpretation. H.‐B. Leu, and Y.‐L. Yang contributed to paper writing. All the investigators approved the paper.

## Supporting information

Supporting information.

Supporting information.
