# Supplementary material for: Optimal blood pressure for patients with end‐stage renal disease following coronary interventions
Source: J Clin Hypertens (Greenwich). 2021 Jul 15;23(8):1622–30. doi: 10.1111/jch.14325 (PMC8678782; doi:10.1111/jch.14325)
Supplement: Supplementary file 1 — Supporting information. [file JCH-23-1622-s001.docx]

Supplement table 1. Clinical outcome in ESRD patients according to systolic and diastolic blood pressure

|  |  | Systolic BP |  |  |  | Diastolic BP |  |
| --- | --- | --- | --- | --- | --- | --- | --- |
|  |  | Crude HR | Adjusted HR |  |  | Crude HR | Adjusted HR |
| Cardiac death | ＜ 120 mmHg | 4.98 (1.52-16.30) | 4.13 (1.25-13.63) |  | < 70 mmHg | 7.28 (1.77-29.9) | 4.60(1.09-19.42) |
|  | 120-129 mmHg | 3.13 (0.89-10.97) | 2.77 (0.78-9.77) |  | 70-79 mmHg | 3.33(0.76-14.58) | 2.70(0.61-11.94) |
|  | 130-139 mmHg | 2.31 (0.612-8.69) | 2.79 (0.74-10.52) |  | 80-89 mmHg | Referent | Referent |
|  | 140-149 mmHg | Referent | Referent |  | ≧ 90 mmHg | 4.47(0.90-22.16) | 4.02(0.81-22.11) |
|  | 150-159 mmHg | 2.17(0.54-8.68) | 2.87 (0.71-11.57) |  |  |  |  |
|  | ≧ 160 mmHg | 2.15 (0.58-7.93) | 4.13 (1.25-13.63) |  |  |  |  |
|  |  |  |  |  |  |  |  |
| *Nonfatal MI | ＜ 120 mmHg | 1.49 (0.53-4.19) | 1.63 (0.58-4.62) |  | < 70 mmHg | 0.89 (0.39-2.03) | 1.19 (0.48-2.94) |
|  | 120-129 mmHg | 1.37 (0.47-4.04) | 1.47 (0.50-4.35) |  | 70-79 mmHg | 0.91 (0.39-2.10) | 1.12 (0.47-2.68) |
|  | 130-139 mmHg | 0.69 (0.19-2.57) | 0.65 (0.17-2.43) |  | 80-89 mmHg | Referent | Referent |
|  | 140-149 mmHg | Referent | Referent |  | ≧ 90 mmHg | 0.96 (0.31-2.93) | 0.86 (0.28-2.63) |
|  | 150-159 mmHg | 1.11 (0.32-3.83) | 0.98 (0.28-3.39) |  |  |  |  |
|  | ≧ 160 mmHg | 1.84 (0.66-5.16) | 1.58 (0.56-4.49) |  |  |  |  |
|  |  |  |  |  |  |  |  |
| *Stroke | ＜ 120 mmHg | 2.71 (0.32-23.25) | 2.49 (0.29-24.47) |  | < 70 mmHg | . | . |
|  | 120-129 mmHg | 2.65 (0.30-23.84) | 2.57 (0.28-23.46) |  | 70-79 mmHg | . | . |
|  | 130-139 mmHg | 1.73 (0.16-19.12) | 2.00 (0.18-2.20) |  | 80-89 mmHg | Referent | Referent |
|  | 140-149 mmHg | Referent | Referent |  | ≧ 90 mmHg | . | . |
|  | 150-159 mmHg | 2.35 (0.21-25.91) | 2.79 (0.25-31.07) |  |  |  |  |
|  | ≧ 160 mmHg | 2.01 (0.21-19.32) | 2.52 (0.26-24.60) |  |  |  |  |
|  |  |  |  |  |  |  |  |
| *Heart failure | ＜ 120 mmHg | 0.74 (0.34-1.62) | 0.69 (0.31-1.51) |  | < 70 mmHg | 1.66 (0.77-3.56) | 0.99 (0.45-2.23) |
|  | 120-129 mmHg | 0.54 (0.23-1.31) | 0.53 (0.22-1.29) |  | 70-79 mmHg | 1.47 (0.67-3.23) | 1.09 (0.49-2.46) |
|  | 130-139 mmHg | 1.11 (0.50-2.24) | 1.22 (0.55-2.71) |  | 80-89 mmHg | Referent | Referent |
|  | 140-149 mmHg | Referent | Referent |  | ≧ 90 mmHg | 2.53 (1.04-6.20) | 2.46 (1.00-6.04) |
|  | 150-159 mmHg | 1.45 (0.66-3.20) | 1.46 (0.66-3.23) |  |  |  |  |
|  | ≧ 160 mmHg | 1.35 (0.65-2.80) | 1.49 (0.71-3.11) |  |  |  |  |

Major adverse cardiac Event (MACE) includes cardiac death, nonfatal MI, nonfatal stoke; Total major events includes MACE plus hospitalization for CHF

* Nonfatal MI: nonfatal myocardial infraction; stroke: nonfatal stroke; Heart failure: Hospitalization for heart failure.

**Adjusted with age and gender, history of hypertension, diabetes, and smoking
