# Supplementary material for: Optimal blood pressure for patients with end‐stage renal disease following coronary interventions
Source: J Clin Hypertens (Greenwich). 2021 Jul 15;23(8):1622–30. doi: 10.1111/jch.14325 (PMC8678782; doi:10.1111/jch.14325)
Supplement: Supplementary file 2 — Supporting information. [file JCH-23-1622-s002.docx]

Supplement table 2. Age and sex subgroup analysis in chronic renal disease patients according to systolic and diastolic blood pressure

|  |  | Systolic BP |  |  |  | Diastolic BP |  |
| --- | --- | --- | --- | --- | --- | --- | --- |
|  |  | Crude HR | Adjusted HR |  |  | Crude HR | Adjusted HR |
| PCI at age <70 |  |  |  |  |  |  |  |
| MACE | ＜ 120 mmHg | 2.01 (0.74-5.49) | 1.84 (0.62-5.19) |  | < 70 mmHg | 2.46 (1.04-5.83) | 2.04 (0.81-5.10) |
|  | 120-129 mmHg | 1.64 (0.57-4.74) | 1.61 (0.54-4.82) |  | 70-79 mmHg | 1.71 (0.72-4.04) | 1.54 (0.64-3.74) |
|  | 130-139 mmHg | 0.41 (0.10-1.70) | 0.42 (0.10-1.80) |  | 80-89 mmHg | Referent | Referent |
|  | 140-149 mmHg | Referent | Referent |  | ≧ 90 mmHg | 1.50 (0.55-4.15) | 1.37 (0.49-3.81) |
|  | 150-159 mmHg | 0.92 (0.27-3.17) | 0.92 (0.26-3.17) |  |  |  |  |
|  | ≧ 160 mmHg | 1.65 (0.60-4.53) | 1.63 (0.59-4.53) |  |  |  |  |
|  |  |  |  |  |  |  |  |
| Total CV event | ＜ 120 mmHg | 2.31 (0.86-6.22) | 2.18 (0.89-6.04) |  | < 70 mmHg | 1.80 (0.88-3.70) | 1.49 (0.69-3.20) |
|  | 120-129 mmHg | 2.17 (0.78-6.03) | 2.16 (0.76-6.19) |  | 70-79 mmHg | 1.50(0.74-3.03) | 1.36 (0.66-2.81) |
|  | 130-139 mmHg | 0.99 (0.31-3.12) | 1.05 (0.33-3.45) |  | 80-89 mmHg | Referent | Referent |
|  | 140-149 mmHg | Referent | Referent |  | ≧ 90 mmHg | 2.17 (1.01-4.67) | 1.94 (0.89-4.20) |
|  | 150-159 mmHg | 2.00 (0.68-5.87) | 1.95 (0.66-5.70) |  |  |  |  |
|  | ≧ 160 mmHg | 2.70 (1.10-7.10) | 2.58 (0.97-6.86) |  |  |  |  |
| PCI at age≧ 70 |  |  |  |  |  |  |  |
| MACE | ＜ 120 mmHg | 4.54 (1.38-14.92) | 4.20 (1.27-13.85) |  | < 70 mmHg | 1.94 (0.60-6.22) | 1.94 (0.60-6.31) |
|  | 120-129 mmHg | 3.29 (0.95-11.48) | 2.91 (0.83-10.21) |  | 70-79 mmHg | 1.31 (0.38-4.49) | 1.37 (0.40-4.76) |
|  | 130-139 mmHg | 3.64 (1.00-13.24) | 3.97 (1.09-14.47) |  | 80-89 mmHg | Referent | Referent |
|  | 140-149 mmHg | Referent | Referent |  | ≧ 90 mmHg | 3.22 (0.72-14.38) | 2.69 (0.59-12.24) |
|  | 150-159 mmHg | 3.24 (0.84-12.54) | 3.95 (1.01-15.48) |  |  |  |  |
|  | ≧ 160 mmHg | 2.71 (0.7-10.21) | 2.77 (0.71-2.62) |  |  |  |  |
|  |  |  |  |  |  |  |  |
| Total CV event | ＜ 120 mmHg | 1.48 (0.77-2.83) | 1.36 (0.71-2.62) |  | < 70 mmHg | 1.65 (0.67-4.09) | 1.45 (0.58-3.65) |
|  | 120-129 mmHg | 0.92 (0.44-1.93) | 0.86 (0.41-1.81) |  | 70-79 mmHg | 1.22 (0.47-3.18) | 1.10 (0.42-2.90) |
|  | 130-139 mmHg | 1.56 (0.75-3.27) | 1.71 (0.81-3.58) |  | 80-89 mmHg | Referent | Referent |
|  | 140-149 mmHg | Referent | Referent |  | ≧ 90 mmHg | 3.33 (1.02-10.93) | 2.86 (0.86-9.47) |
|  | 150-159 mmHg | 1.28 (0.57-2.90) | 1.34 (0.58-3.07) |  |  |  |  |
|  | ≧ 160 mmHg | 1.40 (0.66-2.95) | 1.45 (0.68-3.10) |  |  |  |  |
|  |  |  |  |  |  |  |  |

Major adverse cardiac Event (MACE) includes cardiac death, nonfatal MI, nonfatal stoke; Total major events includes MACE plus hospitalization for CHF

* Nonfatal MI: nonfatal myocardial infraction; stroke: nonfatal stroke; Heart failure: Hospitalization for heart failure.

**Adjusted with age and gender, history of hypertension, diabetes, and smoking
